# Supplementary material for: Serotonin improves behavioral contrast sensitivity of freely moving rats
Source: PLoS One. 2020 Mar 19;15(3):e0230367. doi: 10.1371/journal.pone.0230367 (PMC7082012; doi:10.1371/journal.pone.0230367)
Supplement: Attachment — Submitted filename: Response to Reviewers_SatoYA.docx [file pone.0230367.s001.docx]

Professor Judith Homberg

Academic Editor

PLOS ONE

**Re:** **PONE-D-19-31125**

Serotonin improves behavioral contrast sensitivity of freely moving rats

PLOS ONE

Dear Professor Homberg,

Thank you very much for sending us your decision and the reviewer's comments on our manuscript, "Serotonin improves behavioral contrast sensitivity of freely moving rats" by Sato et al. We appreciate the positive comments and suggestions that have helped improve our study. Accordingly, we have revised our manuscript. In the process of the revision, we found that the population data of water intake and open field test contained one rat which was used in a preliminary experiment to determine their experimental conditions and whose contrast sensitivity was not measured. Therefore, we removed data of the rat from the population data of water intake and open field, by which the number of animals became equally seven in contrast sensitivity and water intake and open field. The statistical significance of the data was not changed after this treatment. Accordingly, the relevant part of text and Fig 4 have been modified.

Details of our responses to the reviewer’s comments are as follows.

**Responses to Journal requirements**

**1. Please ensure that your manuscript meets PLOS ONE's style requirements, including those for file naming. The PLOS ONE style templates can be found at http://www.plosone.org/attachments/PLOSOne_formatting_sample_main_body.pdf and http://www.plosone.org/attachments/PLOSOne_formatting_sample_title_aut
hors_affiliations.pdf**

We have confirmed The PLOS ONE style templates and modified our manuscript to meet the style requirements.

**2. We note that you have stated that you will provide repository information for your data at acceptance. Should your manuscript be accepted for publication, we will hold it until you provide the relevant accession numbers or DOIs necessary to access your data. If you wish to make changes to your Data Availability statement, please describe these changes in your cover letter and we will update your Data Availability statement to reflect the information you provide.**

We have uploaded our data to figshare, and the DOI is as follows;

DOI: 10.6084/m9.figshare.11845611

**3. Thank you for stating the following in the Acknowledgments Section of your manuscript:**

**"This work was supported by KAKENHI 22500573, 216 25282216, 25560302, and 16H01869 to S.S."**

**We note that you have provided funding information that is not currently declared in your Funding Statement. However, funding information should not appear in the Acknowledgments section or other areas of your manuscript. We will only publish funding information present in the Funding Statement section of the online submission form.**

**Please remove any funding-related text from the manuscript and let us know how you would like to update your Funding Statement. Currently, your Funding Statement reads as follows:**

**"The author(s) received no specific funding for this work.**

We have removed the text related funding information in the Acknowledgments section of the revised version of our manuscript. We would like to update the financial disclosure section with the following funding information; “This work was supported by KAKENHI JP22500573, JP25282216, JP25560302, and JP16H01869 to S.S, and JP17J08499 to A.Y.S.”

**4. Please ensure that you refer to Figure 3 in your text as, if accepted, production will need this reference to link the reader to the figure.**

**[Note: HTML markup is below. Please do not edit.]**

We have specified reference to Figure 3 on line 177 of the revised manuscript.

**Responses to the Reviewer1**

We would like to express our thanks to this reviewer for his insightful comments that have very much helped us improve the paper.

***To the reviewer’s comments***

**1. Information about that FLX directly acts on the 5-HT receptor.**

Reviewer 1 suggested discussing the activation 5-HT receptor by FLX. We have discussed this point in the Discussion section (**line 236-242 in the manuscript**).

**2. Information about projection of dorsal raphe nucleus (DR) and median raphe nucleus (MR).**

Reviewer 1 suggested discussing serotonin endogenously released from not only dorsal but also median raphe nucleus. We have discussed this point in the Discussion section (**line 224-228 in the manuscript**).

Again, we would like to thank you, Prof. Judith Homberg, and the reviewers for your time and feedback. We hope the revised manuscript will be now considered suitable for publication in PLOS ONE.

With best wishes,

Satoshi Shimegi
